# Sall4 and Myocd Empower Direct Cardiac Reprogramming From Adult Cardiac Fibroblasts After Injury

**DOI:** 10.3389/fcell.2021.608367

**Published:** 2021-02-26

**Authors:** Hong Zhao, Yi Zhang, Xiaochan Xu, Qiushi Sun, Chunyan Yang, Hao Wang, Junbo Yang, Yang Yang, Xiaochun Yang, Yi Liu, Yang Zhao

**Affiliations:** ^1^State Key Laboratory of Natural and Biomimetic Drugs, The Ministry of Education (MOE) Key Laboratory of Cell Proliferation and Differentiation, Beijing Key Laboratory of Cardiometabolic Molecular Medicine, College of Future Technology, Institute of Molecular Medicine, Peking University, Beijing, China; ^2^PKU-Nanjing Institute of Translational Medicine, Nanjing, China; ^3^Beijing Key Lab of Traffic Data Analysis and Mining, School of Computer and Information Technology, Beijing Jiaotong University, Beijing, China; ^4^The Center for Models of Life, Niels Bohr Institute, Copenhagen, Denmark; ^5^Peking-Tsinghua Center for Life Sciences, Peking University, Beijing, China; ^6^Plastech Pharmaceutical Technology Co., Ltd., Nanjing, China

**Keywords:** myofibroblast, cardiac reprogramming, high efficiency, Sall4, Myocd, MICF

## Abstract

Direct conversion of fibroblasts into induced cardiomyocytes (iCMs) holds promising potential to generate functional cardiomyocytes for drug development and clinical applications, especially for direct *in situ* heart regeneration by delivery of reprogramming genes into adult cardiac fibroblasts in injured hearts. For a decade, many cocktails of transcription factors have been developed to generate iCMs from fibroblasts of different tissues *in vitro* and some were applied *in vivo*. Here, we aimed to develop genetic cocktails that induce cardiac reprogramming directly in cultured cardiac fibroblasts isolated from adult mice with myocardial infarction (MICFs), which could be more relevant to heart diseases. We found that the widely used genetic cocktail, Gata4, Mef2c, and Tbx5 (GMT) were inefficient in reprogramming cardiomyocytes from MICFs. In a whole well of a 12-well plate, less than 10 mCherry^+^ cells (<0.1%) were observed after 2 weeks of GMT infection with *Myh6*-reporter transgenic MICFs. By screening 22 candidate transcription factors predicted through analyzing the gene regulatory network of cardiac development, we found that five factors, GMTMS (GMT plus Myocd and Sall4), induced more iCMs expressing the cardiac structural proteins cTnT and cTnI at a frequency of about 22.5 ± 2.7% of the transduced MICFs at day 21 post infection. What is more, GMTMS induced abundant beating cardiomyocytes at day 28 post infection. Specifically, Myocd contributed mainly to inducing the expression of cardiac proteins, while Sall4 accounted for the induction of functional properties, such as contractility. RNA-seq analysis of the iCMs at day 28 post infection revealed that they were reprogrammed to adopt a cardiomyocyte-like gene expression profile. Overall, we show here that Sall4 and Myocd play important roles in cardiac reprogramming from MICFs, providing a cocktail of genetic factors that have potential for further applications in *in vivo* cardiac reprogramming.

## Introduction

Cardiovascular disease remains a leading cause of death worldwide, for which current therapeutic methods remain limited. As an attractive approach for heart regeneration, reprogramming fibroblasts into induced cardiomyocytes (iCMs) by the over-expression of transcription factors has been researched for a decade. Ectopic expression of Gata4, Mef2c, and Tbx5 (referred to as GMT), was first reported to initiate cardiac gene expression in mouse cardiac and tail-tip fibroblasts, which could be ultimately reprogrammed to a spontaneously contractile state, albeit at a low efficiency (Ieda et al., 2010). Follow-up papers demonstrated that many factors, such as other transcription factors (Protze et al., 2012), microRNAs (Jayawardena et al., 2012), activated kinase (Zhou et al., 2015), small molecules (Ifkovits et al., 2014; Zhao et al., 2015), and epigenetic factors (Zhou et al., 2016, 2018) could further increase the reprogramming efficiency. Moreover, several groups have found that human fibroblasts can be directly reprogrammed toward the cardiac lineage (Fu et al., 2013; Nam et al., 2013; Wada et al., 2013), paving the way to further applications in regenerative medicine.

Besides *in vitro* cardiac reprogramming, direct *in vivo* cardiac reprogramming was first reported in 2012 by three independent groups using different tracing systems (Inagawa et al., 2012; Qian et al., 2012; Song et al., 2012). Although with different reprogramming efficiencies, infarcted mice in the treatment groups resulted in significant improvement of cardiac performance in these studies. Different strategies have been applied, such as small molecules (Mohamed et al., 2017), nanoparticle-based delivery (Chang et al., 2019), and non-integrating Sendai virus (SeV) (Miyamoto et al., 2018), to increase the *in vivo* reprogramming efficiency. By using a tamoxifen-inducible fibroblast-lineage tracing model, *Tcf21*^*iCre*^/ *R26*-tdTomato, Miyamoto et al. (2018) demonstrated that 1 week post injection about 1.5% of tdTomato^+^ cells expressed cTnT in SeV GMT-injected mice whereas only 0.5% of tdTomato^+^ cells expressed cTnT in the retrovirus-treated groups. Even after 4 weeks of induction, the reprogramming efficiency was as low as 5% (Miyamoto et al., 2018). As discussed by Srivastava and Ieda, high reprogramming efficiency depends on many factors, such as the combination of optimal factors, an effective delivery system, and the source of fibroblasts (Srivastava and Ieda, 2012). For cardiac reprogramming, the clinical potential is to induce *in situ* myofibroblasts into cardiomyocytes. However, in most studies, researchers optimized the reprogramming factors by using murine embryonic fibroblasts, tail-tip fibroblasts, or neonatal cardiac fibroblasts (NCFs) as cellular sources (Fu et al., 2015; Mohamed et al., 2017; Guo et al., 2019), which are easier to obtain but less relevant to the target cell type for direct *in situ* cardiac reprogramming. A better choice is to use adult cardiac fibroblasts (ACFs) (Zhao et al., 2015; Zhou et al., 2017), yet they are isolated from mice without cardiac injury. The myofibroblast, playing critical roles in acute healing after myocardial infarction and long-standing fibrosis with chronic disease (Weber et al., 2013), represents the most diseased-related cellular source for direct cardiac reprogramming.

The cardiac myofibroblast exhibits different gene expression profiling: more than one thousand genes are differentially expressed compared with the cardiac resident fibroblast, which is the main origin of cardiac myofibroblast (Kanisicak et al., 2016). Many of these genes are enriched in clusters related to wound healing, extracellular matrix component production, mesenchymal cell differentiation, cell adhesion and fibrotic disease states. Specifically, a major subset of cardiac fibroblasts in the uninjured adult murine heart express the marker gene *Tcf21*. When those fibroblasts are activated and differentiate into myofibroblasts, they down-regulate *Tcf21* expression and up-regulate *Postn* and *Acta2* expression. Moreover, myofibroblasts are heterogeneous according to recently published single-cell RNA sequencing data. Farbehi et al. (2019) described different myofibroblast subpopulations by either profibrotic or antifibrotic signatures. Ruiz-Villalba et al. (2019) revealed that activated fibroblasts exhibit a clear profibrotic signature, express high levels of the hormone Cthrc1, and localize to the injured myocardium. To develop more powerful cocktails for reprogramming, it is necessary to evaluate the reprogramming potential of the cardiac myofibroblast with the delivery of potential reprogramming factors.

In the present study, we sought to directly induce MICF into cardiomyocytes with high efficiency, which is inefficient with only GMT. After screening additional 22 transcription factors, we revealed that GMT plus Myocd and Sall4 (referred to as GMTMS) was able to efficiently generate cardiomyocyte-like cells with functional properties.

## Materials and Methods

### Animals and Surgery

The animal protocol for surgery followed the institutional guidelines and was approved by PKU Institutional Animal Care and Use Committee. *Myh6*-mCherry mice specifically expressing mCherry in cardiomyocytes driven by the mouse *Myh6* promoter, were a gift from Dr. Kotlikoff (Jesty et al., 2012). *Postn*-MCM^+/+^ mice (Kanisicak et al., 2016), a gift from Dr. Molkentin were used to trace myofibroblast by crossing with *Rosa26*-lsl-tdTomato^+/+^ mice (Madisen et al., 2010). Myocardial infarction (MI) surgery was induced by permanent ligation of the left anterior descending artery (LAD) as described (Virag and Lust, 2011). Briefly, mice (8 weeks old) were anaesthetized with 2.5% isoflurane/97.5% oxygen and placed in a supine position. Animals were intubated with a 24 G stump needle and ventilated with 1.5% isoflurane/98.5% oxygen using a VentElite mouse ventilator (Harvard Apparatus); stroke volume, 220 μl; respiratory rate, 120 breaths per min. MI was induced by permanent ligation of the LAD with a 6-0 nylon suture. To activate the inducible MerCreMer protein, tamoxifen (Sigma) was dissolved in corn oil (90%) and ethanol (10%) at a concentration of 20 mg/ml and administered (0.2 mg/g mouse body weight) every other day by oral gavage into *Postn*-MCM^+/–^; *Rosa26*-lsl-tdTomato^+/–^ mice since the surgery day. Non-MI mice were treated with tamoxifen for 7 times as the same way before sacrifice.

### Primary Cells Isolation

NCFs were isolated by enzyme digestion method following with Thy1^+^ sorting by MACS (magnetic-activated cell sorting) as described by Wang et al. (2015b). ACFs from adult mice without injury were isolated using a described method (Zafeiriou et al., 2016). To isolate MICFs, hearts were removed from mice with MI surgery for 3, 5, 7, 10, and 14 days and washed in cold PBS. The infarcted tissue was cut from the whole heart, then minced into small pieces (≤1 mm^3^). Samples from each heart were digested in 2 ml collagenase I/II/IV (Gibco, 2 mg/ml) with Dispase II (Sigma, 2 mg/ml) and incubated at 37°C in a cell culture chamber for 2–3 h. After digestion, the cells were passed through a 70 μM filter, centrifuged at 1,100 rpm for 3 min, and then plated in 12-well plates with culture medium (DMEM supplemented with 10% FBS, 1% NEAA, 1% GlutaMax, and 1% penicillin/streptomycin). Adult cardiomyocytes were isolated by a langendorff-free method (Ackers-Johnson et al., 2016).

### Plasmids

**FIGURE 1:**
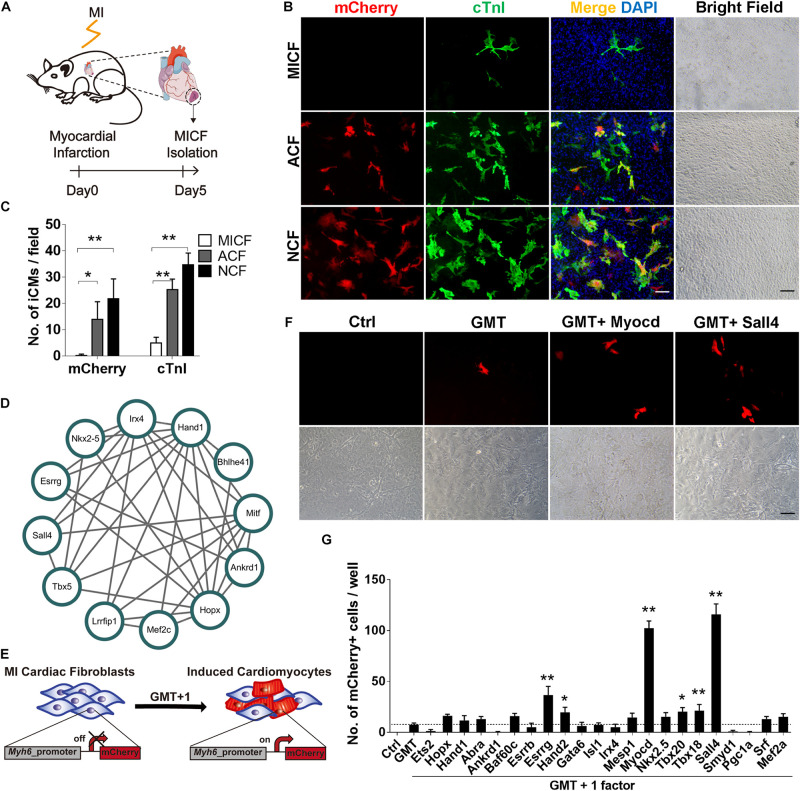
GMT is inefficient to reprogram MICFs toward a cardiac fate and screening for additional factors. **(A)** Flowchart of the strategy to isolate cardiac fibroblats from mice with myocardial infarction (MICFs) **(B)** Immunofluorescent characterization of cardiomyocyte-like cells induced by GMT from different cardiac fibroblasts 3 weeks after transduction. **(C)** Quantification of mCherry^+^ or cTnI^+^ cells induced by GMT (*n* = 4). **(D)** A core regulating network of transcription factors for heart development by weighted correlation network analysis of published scRNA-seq data. **(E)** Schematic of screening candidate transcription factors. **(F)** Myocd or Sall4 increased the number of *Myh6*-mCherry positive cells. Photographed 2 weeks after induction. **(G)** Quantitative data of mCherry^+^ cells induced by GMT plus another factor at day 14 (*n* = 3). All data are presented as mean ± SD. *p < 0.05; **p < 0.01 vs. relevant control. Scale bars: 100 μm.

Lentiviral constructs were generated by subcloning. First, CDS sequences of Gata4, Mef2c, and Tbx5 or the polycistronic cassette MGT from pMX vectors were amplified by PCR and then cloned into the Fu-tet-O vector (Addgene, #19778) through Gibson ligation according to the manufacturer’s instructions (TransGene). Retroviral vectors encoding Gata4, Mef2c, and Tbx5 in the pMXs vector were as described previously (Ieda et al., 2010). The polycistronic pMX-MGT was generously provided by Dr. Qian (Wang et al., 2015a). The lentivirus packaging and envelope vectors, psPAX2 (Addgene, #12260) and pMD2.G (Addgene, #12259), were from Addgene. To compare the transduction efficiency, retro- or lenti-virus described above was infected into MICFs. We achieved higher infection efficiency using the Tet-On system, which was used in subsequent experiments and applied to the construction of other 22 transcription factors including Myocd and Sall4 (Figure 1G). Note that CDS sequences of BAF60C, ESRRG, SMYD1, and SRF were derived from human.

### Viral Packaging

Retrovirus packaging was performed in platE cells, and lentivirus packaging was conducted in 293T cells. Both of these cell lines were maintained in high glucose DMEM growth medium (Hyclone) containing 10% FBS, 1% GlutaMax, and 1% NEAA. One day before transfection, platE and 293T cells were plated in 10 cm dishes. The next day, platE cells were transfected with 20 μg pMXs-based retrovirus vectors for retrovirus packaging using 60 μl PEI according to the manufacturer’s instructions. For lentivirus packaging, 10 μg Fu-tet-O vector, 10 μg psPAX2, and 5 μg pMD2.G were introduced into 293T cells using 75 μl PEI. After 12 h of transient transfection, medium was changed to 10 ml of fresh medium. Supernatant was collected 48 h post transfection and filtered through 45 μm filters. Viruses were frozen at −80°C for future use.

### Induced Cardiomyocyte (iCM) Reprogramming

Isolated cells were plated into 12 well plates. Viruses were added to the culture medium when cells were at a density of about 1.5 × 10^5^ cells per well. Twenty-four hours later, the viral medium was replaced with conditional medium containing DMEM/M199 (4:1) supplemented with 10% FBS, 10% KSR, 1% NEAA, 1% Glutamax, 1% PS, and so on. Doxycycline (1 μg/ml) was also added to the iCM-inducing medium, which was changed every 3 days.

### Flow Cytometry

Several weeks after viral transduction, reprogrammed cells were dissociated with incubation in enzymes. First, with 0.25% Trypsin/EDTA at 37°C for 5–7 min, cells together with a large amount of ECMs were removed from the plate. Then, collagenases I/II/IV (2 mg/ml) were added to the well and incubated at 37°C for 30–40 min. To calculate the proportion of *Postn*-tdTomato^+^ myofibroblasts, MICFs were digested by 0.25% Trypsin/EDTA at 37°C for 5–7 min. By passing through 40 μm cell strainers, dissociated cells were analyzed on a CytoFlex (Beckman Coulter) or were sorted by Aria3 (BD).

### Immunofluorescence

Cells were fixed in 4% paraformaldehyde for 15 min, permeabilized with PBS buffer containing 0.2% Triton-X for 30 min and blocked with 3% normal donkey serum for 2 h at room temperature. Then, cells were incubated with primary antibodies against Vimentin (Abcam, ab92547, 1:400), Pdgfrα (R&D, AF1062, 1:50), αSMA (Abcam, ab7817, 1:400), Gata4 (Santa Cruz, sc-25310, 1:100), Mef2c (CST, 5030s, 1:400), Tbx5 (Invitrogen, 42-6500, 1:200), cTnT (Thermo Fisher Scientific, MA5-12960, 1:200), cTnI (Abcam, ab56357, 1:300), and α-Actinin (Sigma, A7311, 1:500) at 4°C overnight. The next morning, after washing with PBS, Alexa fluorogenic secondary antibodies (Invitrogen, 1:1,000) were incubated for 1 h at room temperature to detect a signal. DAPI (1: 10,000) was finally used to determine the nuclear localization. Images were captured by inverted fluorescence microscopes from Zeiss (AXIO Vert.A1) and Nikon (Ti-E).

### qRT-PCR

Total RNA was extracted from cells according to the manufacturer’s protocol (TransGene, ET111). cDNA was synthesized using a reverse transcription kit (AE311) from TransGene. Quantitative RT-PCR was performed using a Real-Time PCR system (q225, Kubo Tech) and a SYBR PCR Master Mix (Vazyme, Q311) according to the manufacturer’s instructions. The primer sequences are provided in the Supplementary.

### Ca^2+^ Imaging

To record calcium transients, Ca^2+^ imaging was performed according to the manufacturer’s protocol (Beyotime, S1060). Briefly, GMTMS-induced iCMs at day 28 were incubated with 2 μM Fluo-4 AM in PBS solution at 37°C for 30 min then the cells were washed to de-esterify the Fluo-4 AM for 30 min. Ca^2+^ imaging was performed using a two-photon microscope (Olympus, FVMPE-RS).

### Processing and Analysis of Published scRNA Data

Single cell RNA sequencing data were derived from GSE76118 and cardiac single-cell transcriptome data were collected on days 8.5, 9.5, and 10.5 of embryonic development (Li et al., 2016). Core transcription factors related to 233 tissues and cell types were selected (D’Alessio et al., 2015) and all single-cell data of these candidate TFs were screened from the downloaded RNA-Seq data. After zero-expression and low-expression TFs were filtered out, 1258 TFs were selected as research objects. Analysis was performed in gene expression units of log2 (CPM+1). First, we built a co-expression network of 1258 TFs by WGCNA (Langfelder and Horvath, 2008). Then, we used the Cytoscape plugin cytoHubba (Chin et al., 2014) to extract the key TFs from the co-expression network. Finally, we constructed a cluster of interacting TFs related to heart development.

### Whole Transcriptome Assay

To compare gene expressing profiles of different cardiac fibroblasts, we analyzed MICFs with the CPM data of Sham P1 fibroblasts and Sham P56 fibroblasts from GSE95755 (Quaife-Ryan et al., 2017). The read count data of MICFs was transformed into CPM data for fold change analysis. Hierarchical clustering was performed with 2,585 genes which log2 (fold change) > 2 and GO enrichment analysis was performed using the OmicShare tools^1^.

After a 4 week induction, *Myh6*-mCherry^+^ iCMs from GMTMS- and GMTMM-transduced MICFs were collected by FACS (BD Aria3). RNA was extracted using an RNA Isolation Kit (TransGene, ET111). RNA was sequenced by PE150. For data analysis, data processing was performed with MatLab 2019a. FPKM was normalized with the “quantilenorm” function over the 3 samples and transferred as log2 (FPKM+1). Hierarchical clustering of genes and samples was performed after genes that varied less than 1 through the 3 samples were filtered out. GO enrichment was analyzed with the website http://geneontology.org/. Principal component analysis (PCA) was performed using RNA sequencing data of GSE95755 including fibroblasts and cardiomyotytes from both neonatal and adult mice (Quaife-Ryan et al., 2017). All data is normalized and top 1,000 differentially expressed genes enriched for GO analysis above are used of PCA input.

### Statistical Analyses

All data are expressed as mean ± SD. Student’s *t*-test was used to determine significance of differences between two groups. One-way ANOVA was used to determine the significance of differences when more than 2 groups were compared. Data were analyzed using GraphPad Prism 7 (GraphPad Software, Inc.). *P* < 0.05 was considered statistically significant.

## Results

### GMT Is Inefficient in Reprogramming MICFs Into Cardiomyocytes

To obtain enough MICFs for reprogramming, we first developed an easy protocol to effectively isolate cardiac fibroblasts from mice with acute myocardial infarction surgery (Figure 1A). We isolated MICFs from mice with MI for 3, 5, 7, 10, and 14 days, and acquired the largest amount of MICFs from infarcted mice at day 5 or 7 (Supplementary Figure 1). Flow cytometry analysis revealed that the proportion of tdTomato^+^ myofibroblasts increased to a peak at MI Day 5 (Supplementary Figure 2). In consistence, Dr. Molkentin and collogues reported that the quantity of myofibroblasts in differentiated state with high levels of αSMA was maximal after MI injury for 4–7 days, and those myofibroblasts stopped proliferating and lost αSMA expression by day 10 and onwards within the scar region (Fu et al., 2018). Therefore, we isolated MICFs from infarcted mice at day 5 for the following reprogramming experiments. Consistent with reported *in vivo* results (Kanisicak et al., 2016), we found that about 98% of the isolated cells were Vimentin-positive and approximately 50% were Pdgfrα-positive (Supplementary Figure 3).

We isolated cells from *Myh6*-mCherry transgenic mice and examined whether GMT, shown previously to direct the cardiac reprogramming of murine embryonic fibroblasts, NCFs, and ACFs, was able to reprogram MICFs toward a cardiac cell fate. As positive controls, NCFs and ACFs were also infected with GMT. For NCFs and ACFs reprogramming, many mCherry^+^ cells were observed after a 3 week induction and some mCherry-negative cells also expressed cardiac troponin I (cTnI) and cardiac troponin T (cTnT) in the two kinds of fibroblasts (Figures 1B,C and Supplementary Figure 4). However, for MICFs, only about 0.1% cells were induced into cardiomyocyte-like cells after the infection of Gata4, Mef2c, and Tbx5 (Figures 1B,C and Supplementary Figure 4). To ensure enough starting cells with high-level expression of reprogramming factors, all viruses were titered before use by immunofluorescence assay (Supplementary Figure 5). These data suggested that GMT is inefficient for reprogramming iCMs from MICFs, in comparison with NCFs and ACFs.

By comparing gene expressing profiles between MICFs and published ACFs as well as NCFs (Quaife-Ryan et al., 2017), we found more than 2,000 genes were differently expressed in MICFs, in consistent with the previous report (Fu et al., 2018). Many up-regulated genes involved in clusters such as immune process, inflammatory response, extracellular matrix, cell proliferation, cell adhesion and migration (Supplementary Figure 6). The apparent different gene expression profiling between ACFs and MICFs further supported the difference in their potential for cardiac reprogramming.

### Screening for New Factors to Improve Reprogramming Efficiency From MICFs

To identify optimal factors for successful reprogramming of MICFs, we constructed a small library consisting of 22 transcription factors, which are important for heart development. First, we analyzed single-cell RNA sequencing data of cardiac cells in the embryonic period (Li et al., 2016) and constructed a core transcription factor regulating network for heart development by weighted correlation network analysis (WGCNA) (Langfelder and Horvath, 2008; Figure 1D). Combining this network with 14 factors that are necessary for heart development and have been screened on NCFs by Ieda et al. (2010), we obtained a list of 25 factors as candidate genes to perform screening experiments (Figure 1E).

**FIGURE 2:**
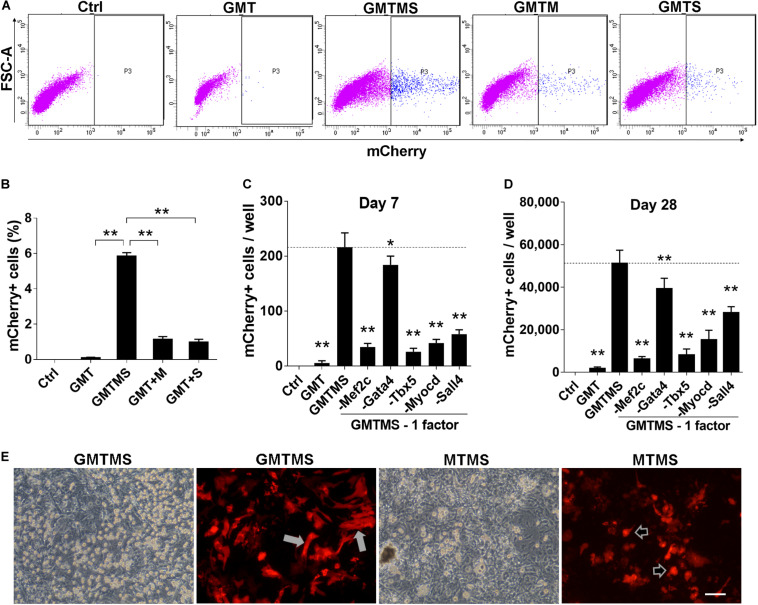
GMTMS significantly enhance the reprogramming efficiency of MICFs toward a cardiomyocyte-like fate. **(A)** Representative flow cytometry plot of *Myh6*-mCherry^+^ cells 2 weeks after induction with indicated factors. **(B)** Summary of FACS analyses for *Myh6*-mCherry expression (*n* = 4). The percentage of mCherry^+^ cells was significantly increased with GMT plus Myocd and Sall4 induction. **(C,D)** Effects of the removal of individual factors from the cocktail on mCherry^+^ cells induction, day 7 and day 28, respectively (*n* = 3). **(E)** Representative iCMs induced by GMTMS or MTMS 4 weeks after transduction. The morphology of mCherry^+^ iCMs tended to be round (shown by arrowheads) rather than rod shape (shown by white arrows) when withdrawing Gata4 from the cocktail. All data are presented as mean ± SD. *p < 0.05; **p < 0.01 vs. relevant control. Scale bars: 100 μm.

After the first round of screening, we found that, compared to GMT alone (less than 10 mCherry^+^ cells per well of a 12-well plate observed under fluorescence microscope, <0.1%), GMT plus Myocd or Sall4 significantly increased the number of mCherry^+^ cells (Figures 1F,G). After the induction of MICFs with GMT plus Myocd and Sall4 (GMTMS) for 14 days, abundant mCherry^+^ cells emerged. Flow cytometry analysis revealed that 5.8% of the MICFs infected with GMTMS expressed mCherry while only 0.1% of those infected with GMT (Figures 2A,B). To identify factors to further improve reprogramming efficiency, we screened the remaining factors for a second round using a plus-one strategy in the presence of GMTMS. However, no more factors further increased the percentage of mCherry^+^ cells (Supplementary Figure 7).

To identify dispensable factors, each factor of GMTMS was individually removed from the medium. We found that removing each one of the factors decreased the numbers of mCherry^+^ cells or beating cells. Although Gata4 seemed to be dispensable according to the number of mCherry^+^ cells (Figures 2C,D), most of these cells tended to be round rather than spindle-shaped without Gata4 infection at a later stage (Figure 2E). Many cells in the GMTMS induction wells appeared thicker on phase-contrast microscopy, with a polygonal or rod-like shape as shown by mCherry fluorescent protein (Figure 2E). Removal of Sall4 failed to induce beating cardiomyocytes. GMT plus Sall4 induced many spontaneously beating iCMs (Supplementary Movies S1, S2). Abundant cells with strong beatings were observed 4 weeks following GMTMS induction (Supplementary Movies S3–S5).

### Characterization of Induced Cardiomyocytes by GMTMS From MICFs

**FIGURE 3:**
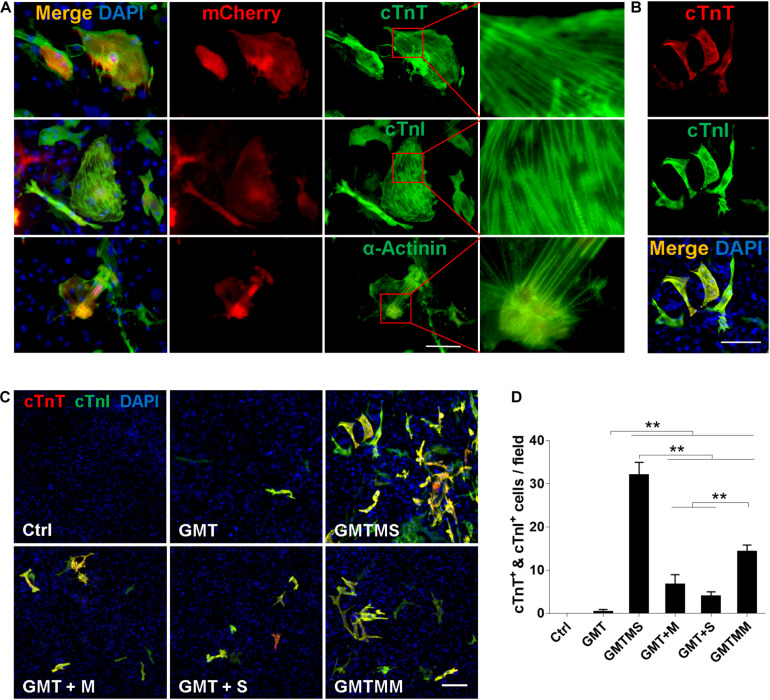
Cardiomyocyte-like cells induced by GMTMS from MICFs express multiple structural proteins. **(A)** Immunostaining for cTnT, cTnI, and α-Actinin at day 21 after GMTMS transduction in MICFs derived from *Myh6*-mCherry mice. mCherry^+^ cells expressed cardiac proteins with well-organized sarcomeric structures. **(B)** Representative images of GMTMS-induced iCMs co-expressing cTnT and cTnI. **(C)** Immunofluorescence for cTnT (red) and cTnI (green) was performed 3 weeks after transduction of MICFs (from C57 mice, 8 weeks old) with GMT, GMTM, GMTS, GMTMS, and GMTMM. **(D)** Quantification of cTnT and cTnI double positive cells (*n* = 3). All data are presented as mean ± SD. **p < 0.01 vs. relevant control. Scale bars: 100 μm.

We performed immunofluorescence assays to determine whether cardiac proteins were expressed after 3 week induction by GMTMS. We found that many mCherry^+^ cells expressed the cardiac structural proteins cTnT, cTnI, and α-Actinin with well-defined sarcomeric structures (Figure 3A). To detect multiple cardiac proteins in single cardiomyocytes, we isolated MICFs from wild type C57 mice and infected the MICFs with different transcription factor cocktails. Compared to the cocktail of Gata4, Mef2c, Tbx5, Myocd, and Mesp1 (GMTMM), which efficiently induced adult human cardiac fibroblasts into iCMs (Wada et al., 2013), GMTMS induced more than twofold of iCMs co-expressing the cardiomyocyte-specific proteins cTnT and cTnI (22.5 ± 2.7% of transduced MICFs) (Figures 3B–D). Moreover, MICFs-derived iCMs exhibited spontaneous Ca^2+^ oscillation after 4 weeks of induction by GMTMS (Supplementary Movie S6). These findings suggested that GMTMS have successfully induced MICFs into iCMs expressing multiple cardiac markers and possessing physiological characteristic of cardiomyocytes.

**FIGURE 4:**
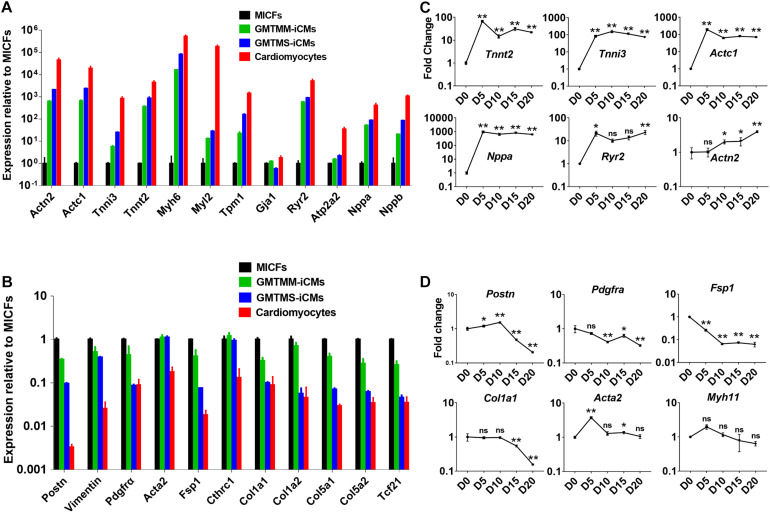
GMTMS up-regulate cardiac genes and down-regulate fibroblast genes during the reprogramming of MICFs toward a cardiac fate. Compared to MICFs, the mRNA expression of cardiac genes was significantly up-regulated **(A)** and the mRNA expression of fibroblast genes was obviously down-regulated **(B)** in GMTMS-induced cardiomyocytes after 4 weeks (*n* = 3). Time course of mRNA expression of cardiac genes **(C)** and fibroblast or smooth muscle genes **(D)** in GMTMS-infected MICFs at early stage, determined by Quantitative RT-PCR (*n* = 3). All data are presented as mean ± SD. *p < 0.05; **p < 0.01 vs. relevant control and ns means no significant difference.

Then, we sorted mCherry^+^ iCMs by FACS at 4 weeks after introducing GMTMS into MICFs and compared the expression of cardiac-specific genes and fibroblast-enriched genes by quantitative RT-PCR. Compared with MICFs, even with GMTMM-induced cardiomyocytes, GMTMS strongly up-regulated the expression of a panel of cardiac genes related to different functions, including sarcomere structure, ion channels, and natriuretic peptides, suggesting a more comprehensive reprogramming by GMTMS (Figure 4A). In contrast, expression of the fibroblast-enriched genes *Postn*, *Pdgfrα*, *Fsp1*, *Tcf21*, and *Collagens* was significantly down-regulated in iCMs induced by GMTMS (Figure 4B). We found variations in the expression levels of different cardiac markers. For example, the structural gene *Tnnt2*, natriuretic peptide *Nppa*, as well as the calcium channel receptor *Ryr2*, were expressed at relative high levels in GMTMS-induced iCMs, whereas the structural gene *Myl2* was expressed at a low level in contrast to cardiomyocytes isolated from adult mice. We also examined the reprogramming kinetics of gene expression at early stage. The cardiac genes *Tnnt2*, *Tnni3*, *Actc1*, and *Nppa* were significantly up-regulated from Day5 and subsequently down-regulated at later stage while the expression of *Actn2* and *Ryr2* was gradually increased with a mild speed (Figure 4C). This reflects the diversity of expressing pattern for different genes during the reprogramming process. The expression of fibroblast genes, *Postn*, *Pdgfrα*, *Fsp1*, and *Col1a1* decreased significantly over time (Figure 4D). However, *Acta2* mRNA expression during reprogramming was at a relatively stable level except the early stage (Figures 4B,D). Similarly, Wada et al. (2013) have reported that the iCMs induced from human cardiac fibroblasts by GMTMM co-express αSMA and α-Actinin. Of note, αSMA is not specific for myofibroblast and it also expresses in embryonic cardiomyocytes (Kruithof et al., 2003). It is possible that the iCMs induced by GMTMS are relatively immature or still maintain the characteristic of myofibroblasts.

**FIGURE 5:**
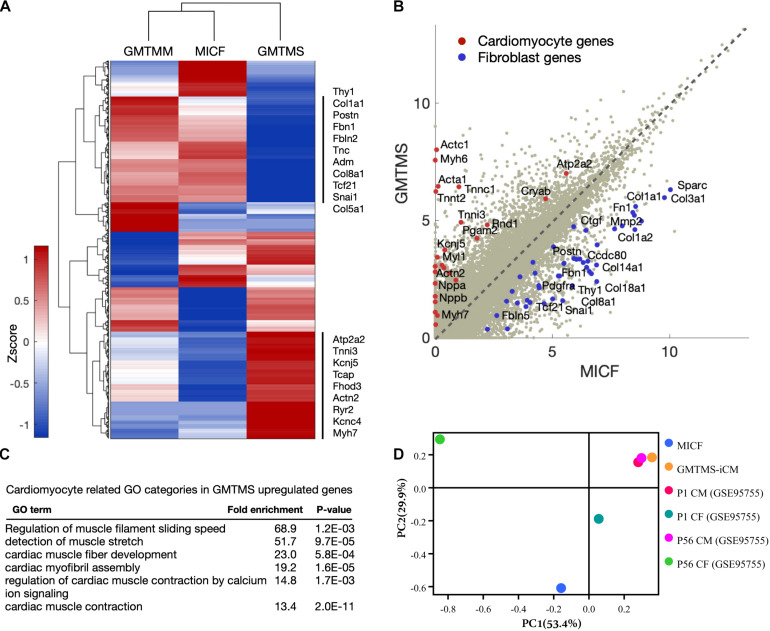
GMTMS-induced iCMs are transcriptionally reprogrammed toward a cardiomyocyte fate. **(A)** Genome-wide comparison between GMTMM and GMTMS on MICF reprogramming. The gene profile was normalized with log_2_(FPKM+1) and then quantile-normalized over multiple samples. Hierarchical clustering was performed with 2,620 genes which varied more than 1 among the 3 samples. **(B)** Scatter plots of gene expression in MICF and GMTMS-iCMs. Gray dots, all of the genes; red dots, cardiomyocyte specific genes; blue dots, fibroblast related genes. **(C)** GO enrichment analysis of top 500 up-regulated genes by GMTMS. Only categories typically related to cardiomyocytes are shown. **(D)** PCA result demonstrates the cell fate of iCMs induced by GMTMS is more closed to cardiomyotytes but far from fibroblasts.

To further examine GMTMS-induced changes in gene expression on the genome-wide scale, we performed RNA-sequencing of MICFs and iCMs induced by GMTMS or GMTMM (FACS by mCherry, 4 week induction). We found significant changes in the global gene expression profiles of MICFs after GMTMS or GMTMM overexpression (Figure 5A). Compared to MICFs, 1318 genes were significantly up-regulated and 1302 genes were significantly down-regulated in GMTMS-induced iCMs, with a false-discovery rate-controlled *p*-value < 0.01. Scatter plots and heatmaps showed the expression of many cardiac genes, and concomitant suppression of fibroblast genes in GMTMS-reprogrammed iCMs (Figure 5B and Supplementary Figure 8). Gene ontology (GO) enrichment analysis revealed that GMTMS-induced gene expression was related to muscle filament sliding speed and cardiac muscle fiber development, as well as muscle stretch and contraction by calcium (Figure 5C). We next compared the similarity of gene expression profile in GMTMS-induced iCMs with published RNA-seq data of cardiomyotes and fibroblasts (Quaife-Ryan et al., 2017). Principal component analysis revealed that on the whole, the cell fate of iCMs induced by GMTMS was far from both neonatal and adult cardiac fibroblasts but was closed to cardiomyocytes (Figure 5D). These results demonstrated a cardiac-like phenotype reprogrammed from MICFs by GMTMS. However, some cardiac genes were not up-regulated in response to GMTMS induction as well, indicating that the cardiac-like phenotype is incomplete and likely variable between cells in the population.

### Myocd and Sall4 Enhanced Cardiac Reprogramming Efficiency From Both Activated and Inactivated Cardiac Fibroblasts

Next, we investigated whether GMTMS could also increase the cardiac reprogramming efficiency of activated fibroblasts or myofibroblasts, which play critical roles in acute healing after myocardial infarction and long-standing fibrosis with chronic disease (Weber et al., 2013). Traditionally, injury activated fibroblasts were referred to as myofibroblasts, in part because they express contractile genes such as *Acta2* (αSMA) (Tallquist and Molkentin, 2017). However, the MICFs used above for cardiac reprogramming were heterogeneous, including both activated myofibroblasts and inactivated resident cardiac fibroblasts. After a systematic evaluation of the isolated fibroblasts, we found that the percentage of αSMA-positive cells was more correlated with the *in vitro* culture time rather than injury period (Supplementary Figure 9). Even about 70% of cardiac fibroblasts isolated from non-MI mice are αSMA-positive after 48 h culture *in vitro* (Supplementary Figures 19B,C). These data implied αSMA was not a good marker for discriminating *in vitro* cultured myofibroblasts from adult cardiac fibroblasts.

**FIGURE 6:**
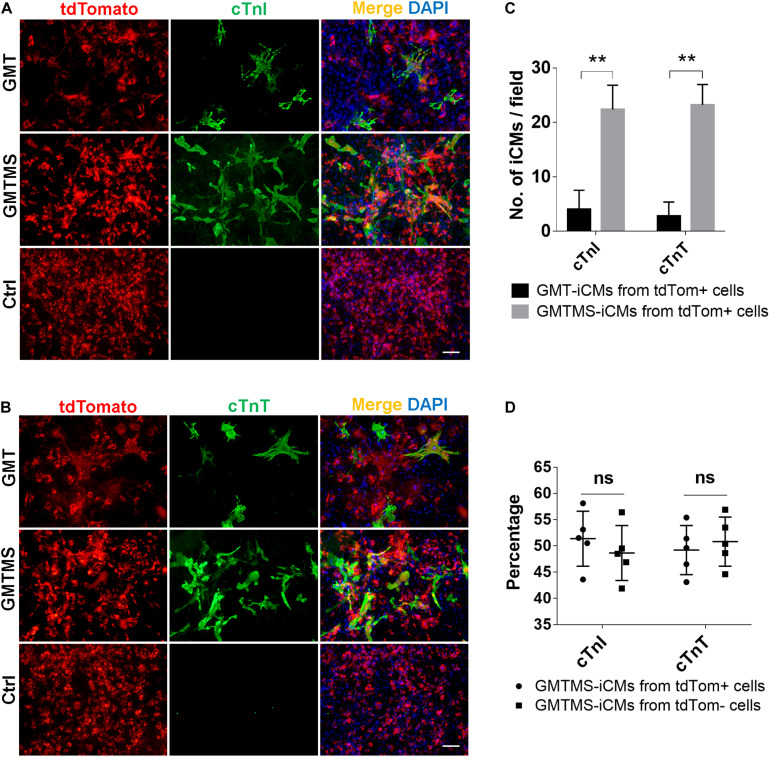
GMTMS significantly increase the iCMs reprogramming efficiency from myofibroblasts traced by *Postn*. **(A,B)** Immunofluorescence for cTnI and cTnT was performed 3 weeks after transduction of indicated cocktails with MICFs (from *Postn*-MCM^+/–^; *Rosa26*-lsl-tdTomato^+/–^ mice, 8 weeks old). **(C)** Quantification of cTnI- or cTnT- positive iCMs induced from *Postn*-tdTomato^+^ myofibroblast by GMTMS and GMT (*n* = 5). **(D)** Quantification of cTnI- or cTnT- positive iCMs induced by GMTMS from *Postn*-tdTomato^+^ myofibroblast or *Postn*-tdTomato^–^ MICFs (*n* = 5). All data are presented as mean ± SD. **p < 0.01 vs. relevant control and ns means no significant difference. Scale bars: 100 μm.

Although the identity and origin of myofibroblasts within the heart have been an ongoing debate, recent lineage tracing studies more faithfully demonstrate that, after injury, *Tcf21*^+^ resident cardiac fibroblasts differentiate into and constitute the majority of disease-activated fibroblasts or myofibroblasts which are *Postn* expressing cells (Moore-Morris et al., 2014; Kanisicak et al., 2016; Kaur et al., 2016). Therefore, we crossed *Postn*-MCM^+/+^ mouse with *Rosa26*-lsl-tdTomato^+/+^ mouse and isolated cardiac fibroblasts from adult offsprings with or without MI surgery. *Postn*-traced tdTomato^+^ myofibroblasts constituted less than 50% of the whole cell populations (Supplementary Figure 2). We found that even by counting tdTomato-positive iCMs, much more iCMs could be induced with GMTMS than that with only GMT after 3 week induction (Figures 6A–C). Actually, there is no significant difference in the numbers of iCMs induced from *Postn*-tdTomato positive and negative cells (Figure 6D).

**FIGURE 7:**
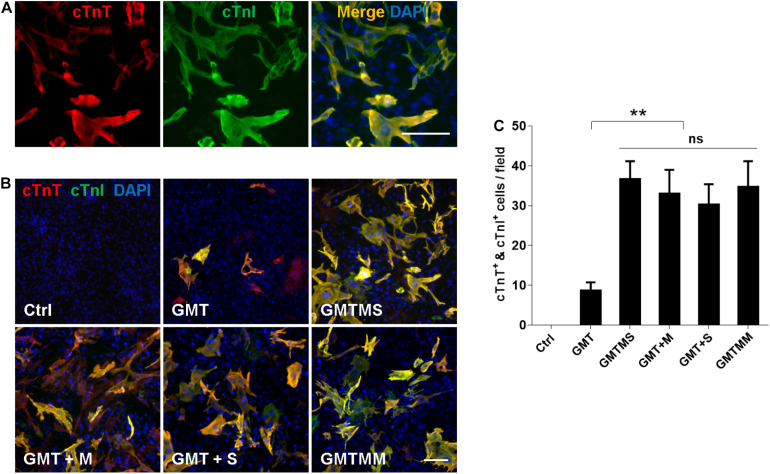
Myocd or Sall4 significantly enhance iCMs reprogramming efficiency from adult cardiac fibroblasts. **(A)** Representative images of GMTMS-induced iCMs co-expressing cTnT and cTnI. **(B)** Immunofluorescence for cTnT (red) and cTnI (green) was performed 3 weeks after transduction of ACFs (from C57 mice without injury, 8 weeks old) with GMT, GMTM, GMTS, GMTMS, and GMTMM. **(C)** Quantification of cTnT and cTnI double positive cells (*n* = 3). Note that four factors (GMTM or GMTS) are enough to reprogram abundant iCMs co-expressing cTnT and cTnI. **p < 0.01 vs. relevant control and ns means no significant difference. Scale bars: 100 μm.

Furthermore, we investigated whether GMTMS could also increase the cardiac reprogramming efficiency from inactivated ACFs isolated from healthy mice. Three weeks after transduction of ACFs with different cocktails, expression of the cardiac markers cTnT and cTnI was tested by immunofluorescence (Figures 7A,B). Consistent with the reprogramming of MICFs and myofibroblasts, GMTMS induced a large number of iCMs, 25.9 ± 3.2% of transduced ACFs, which was also induced by GMTM and GMTS (23.3 ± 4.2% and 21.4 ± 3.6% of transduced ACFs, respectively). There was no significant difference in cTnT- and cTnI-positive iCMs between these groups (Figure 7C). GMT, without additional factors, also induced many cTnT- and cTnI-positive iCMs (6.1 ± 1.5% of transduced ACFs). These suggested that although ACFs were more amenable to reprogramming than MICFs, the additional transcription factors Myocd and Sall4 were also beneficial for their cardiac reprogramming.

## Discussion

Cardiac myofibroblasts are the principal mediators of cardiac fibrosis and scar formation post-MI and are the most prevalent cell type in the injured heart (Weber et al., 2013). Here we evaluated the *in vitro* reprogramming potential of MICFs and demonstrated that MICFs even *Postn*-traced myofibroblasts can be efficiently converted into cardiomyocyte-like cells by the overexpression of five transcription factors including two booster genes, Sall4 and Myocd, revealed in our study.

Myocd (Myocardin) is a transcriptional coactivator of serum response factor (Wang et al., 2001) and is required for vascular smooth muscle (Li et al., 2003) and heart development (Huang et al., 2012). It maintains CM structure and sarcomeric organization, and its cell-autonomous loss in CMs triggers programmed cell death (Huang et al., 2009). Although Myocd promotes the smooth muscle fate in mouse fibroblasts, it appears to activate cardiac rather than smooth muscle gene expression in our study in the context of the other cardiac transcription factors, Indeed, the expression of *Myh11*, a marker for smooth muscle, was extremely low at each reprogramming stage (Figure 4D). Consistent with our results, Nam et al. (2013) reported that overexpression of GATA4, HAND2, TBX5, MYOCD, miR-1, and miR-133 reprogram human fibroblasts into cardiac-like myocytes and Wada et al. (2013) revealed that human fibroblasts can be reprogrammed into iCMs by GATA4, MEF2C, TBX5, MYOCD, and MESP1. In these studies, addition of MYOCD significantly increased the expression of cardiac sarcomeric proteins.

Sall4 is a mammalian Spalt transcription factor expressed by cells of the early embryo and germ cells, an expression pattern similar to other genes associated with early development such as Oct4 and Sox2 (Elling et al., 2006). Sall4 is essential for embryonic stem cell proliferation and widely involved in organ development such as kidney, brain, and heart (Sakaki-Yumoto et al., 2006). Sall4 interacts with Tbx5 to regulate limb and heart patterning in the mouse (Koshiba-Takeuchi et al., 2006) and acts downstream of Tbx5 for pectoral fin outgrowth in the zebrafish (Harvey and Logan, 2006). In our study, by analyzing the gene regulatory network from single cell RNA-sequencing data, we found Sall4 was correlated with the core TFs for the establishment of cardiomyocyte identity. Therefore, it was not quite surprising to find that Sall4 promoted cardiac reprogramming in our study. In cardiac reprogramming from MICFs, we found Sall4 played an important role in inducing spontaneously beating cells, which disappeared solely when Sall4 was withdrawn, indicating Sall4 play roles to fulfill the functional properties of iCMs.

Furthermore, our data showed the difference in cardiac reprogramming between inactivated cardiac fibroblasts and activated myofibroblasts after myocardial infarction. Given myofibroblast is a more appropriate target cell type for *in situ* direct cardiac reprogramming, it is needed to develop reprogramming cocktails directly on myofibroblasts for the purpose of developing cardiac reprogramming cocktails for heart regeneration. Moreover, it could also be intriguing to further study the intrinsic or exogenous factors that determine the differences between ACFs and MICFs (or purified myofibroblasts) in cardiac reprogramming in the future.

## Data Availability Statement

The original RNA-seq data were deposited in the NCBI’s Gene Expression Omnibus database (GEO GSE167436).

## Ethics Statement

The animal study was reviewed and approved by the PKU Institutional Animal Care and Use Committee.

## Author Contributions

YZ, YL, and HZ conceived the project. HZ, CY, HW, JY, YY, and XY performed the experiments. YZ, XX, and QS conducted the bioinformatics analyses. YZ and YL supervised this project. HZ and YZ wrote the manuscript. All authors contributed to the article and approved the submitted version.

## Conflict of Interest

YZ was employed by Plastech Pharmaceutical Technology Co., Ltd. The remaining authors declare that the research was conducted in the absence of any commercial or financial relationships that could be construed as a potential conflict of interest.

## Footnotes

1 http://www.omicshare.com/tools

## Supplementary Material

Supplementary Figure 1 Quantity and morphology of MICFs isolated from mice with MI surgery for 3, 5, 7, 10, and 14 days. MICFs without passaging (P0), 48 h post seeding. Scale bars: 100 μm.

Supplementary Figure 2 Quantification of *Postn*-traced myofibroblasts from MICFs isolated from *Postn*-MCM^+/–^; *Rosa26*-lsl-tdTomato^+/–^ mice. **(A)** Representative flow cytometry plot of *Postn*-tdTomato^+^ myofibroblasts among cardiac firbroblasts isolated from mice with injury or not. **(B)** Quantitative data of *Postn*-tdTomato^+^ myofibroblasts, analyzed by flow cytometry (*n* = 3). All data are presented as mean ± SD. ^∗∗^*p* < 0.01 vs. relevant control.

Supplementary Figure 3 Immunofluorescent characterization of cardiac fibroblasts isolated from mice with MI surgery or not. Representative images of MICFs and ACFs staining with Vimentin **(A)** and Pdgfrα **(B)**, cells without passaging (P0), 48 hours post seeding. Scale bars: 100 μm.

Supplementary Figure 4 Immunofluorescent characterization of cardiomyocyte-like cells induced by GMT from different cardiac fibroblasts. **(A)** Representative images of mCherry^+^ iCMs staining with cTnT. **(B)** Quantification of mCherry^+^ or cTnT^+^ cells induced by GMT (*n* = 4). All data are presented as mean ± SD. ^∗^*p* < 0.05; ^∗∗^*p* < 0.01 vs. relevant control. Scale bars: 100 μm.

Supplementary Figure 5 Infection efficiency of individual lentivirus on MICFs, identified by immunofluorescent assay. Scale bars: 100 μm.

Supplementary Figure 6 Genome-wide comparison between three kinds of cardiac fibroblasts. **(A)** Hierarchical clustering was performed with 2585 differently expressed genes, log2 (fold change) > 2. Up-regulated genes in cluster 1 and 3 mainly related to immune process and inflammatory response, in cluster 5 related to extracellular matrix, cell adhesion and migration and in cluster 7 mainly related to cell proliferation. **(B)** Go enrichment of up-regulated genes in different clusters.

Supplementary Figure 7 Quantitative data of mCherry^+^ cells induced by GMTMS plus another factor at day 14 (*n* = 3). No transcription factor further increased the reprogramming efficiency. All data are presented as mean ± SD. ^∗^*p* < 0.05; ^∗∗^*p* < 0.01 vs. relevant control and ns means no significant difference.

Supplementary Figure 8 Heat maps of cell fate related genes differently expressed between MICF and GMTMS-induced iCM. **(A)** Some fibroblast-enriched genes were down-regulated and **(B)** some cardiomyocyte-specific genes were up-regulated compared with MICFs.

Supplementary Figure 9 The proportion of αSMA-positive cells *in vitro* is correlated with culture time not injury period. Representative images of αSMA-stained MICFs cultured *in vitro* for different times after seeding **(A)**. The proportion of αSMA-positive cells among ACFs and MICFs from mice with different injury days has no significant difference when cultured *in vitro* for 48 h after seeding **(B,C)**. All data are presented as mean ± SD and ns means no significant difference. Scale bars: 100 μm.
