# Supplementary material for: Sall4 and Myocd Empower Direct Cardiac Reprogramming From Adult Cardiac Fibroblasts After Injury
Source: Front Cell Dev Biol. 2021 Feb 26;9:608367. doi: 10.3389/fcell.2021.608367 (PMC7953844; doi:10.3389/fcell.2021.608367)

Suppl. Fig. 1

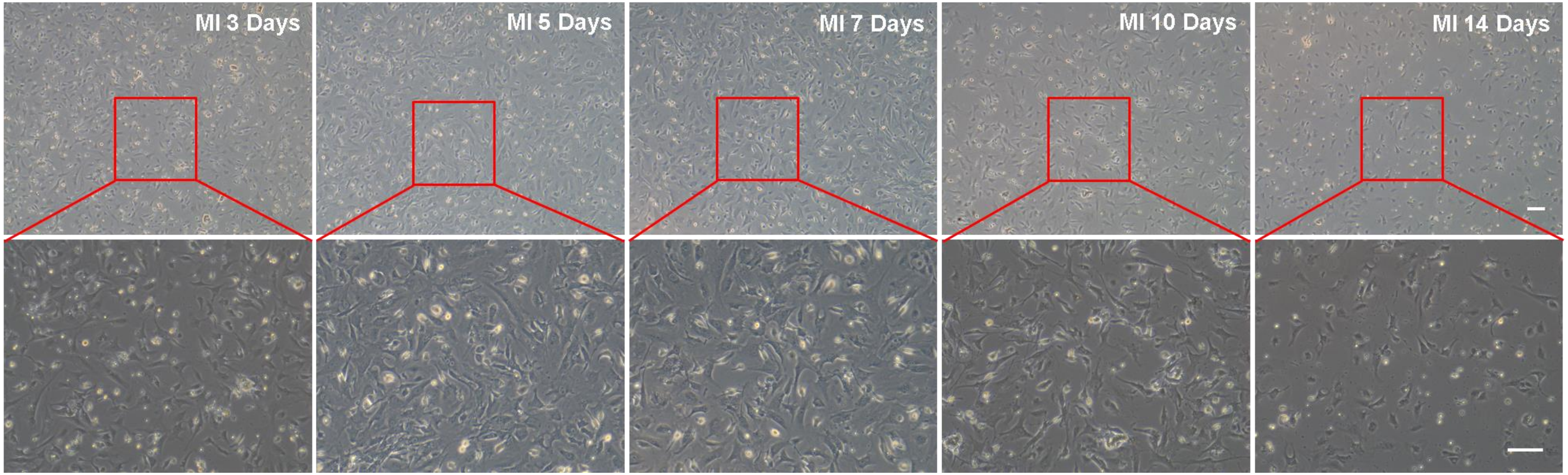

Suppl. Fig. 2A

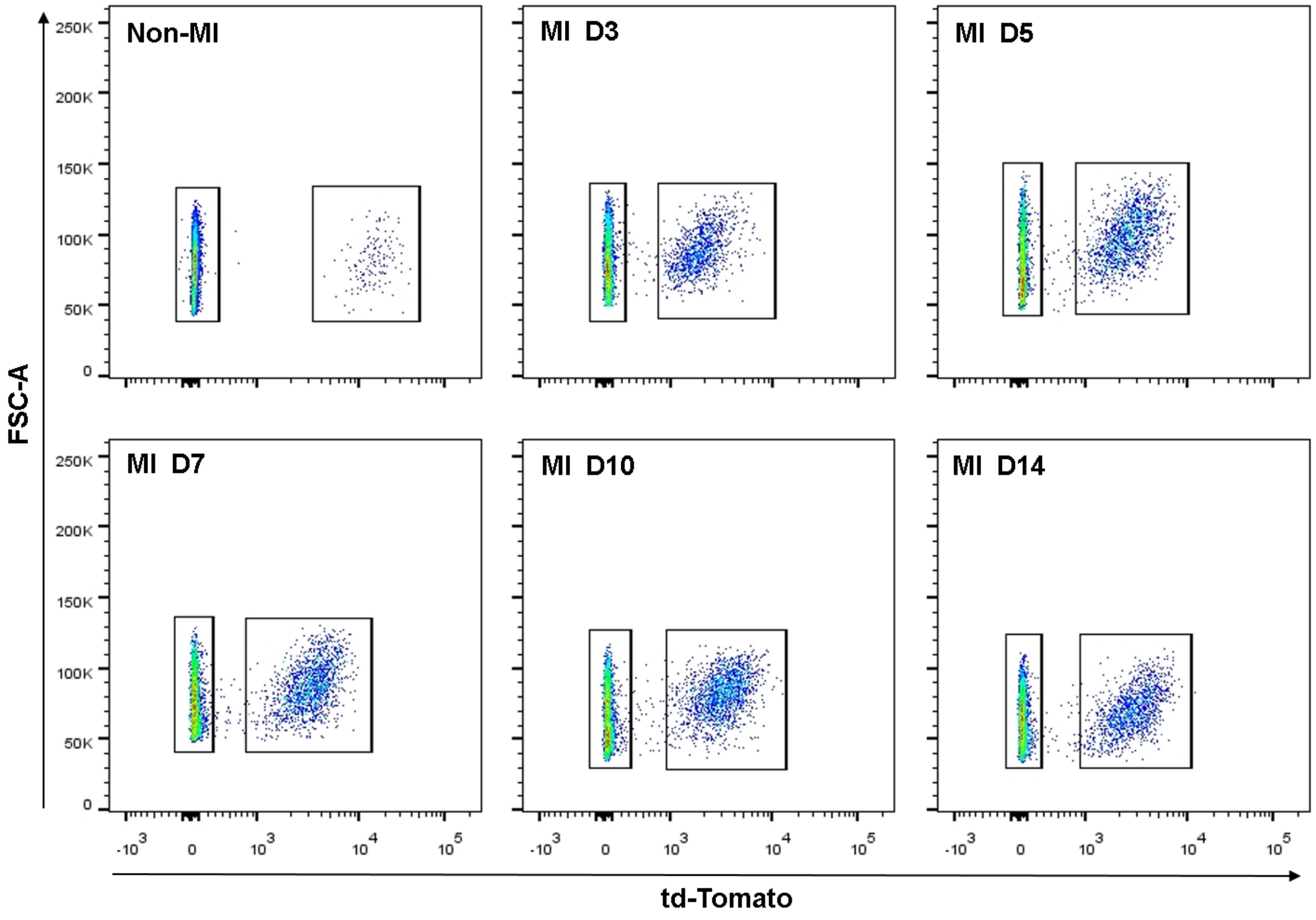

Suppl. Fig. 2B

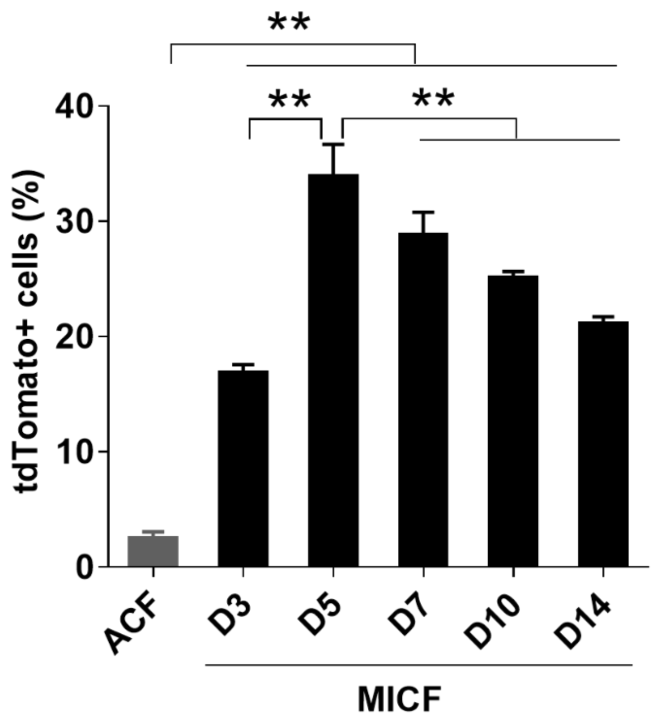

Suppl. Fig. 3A

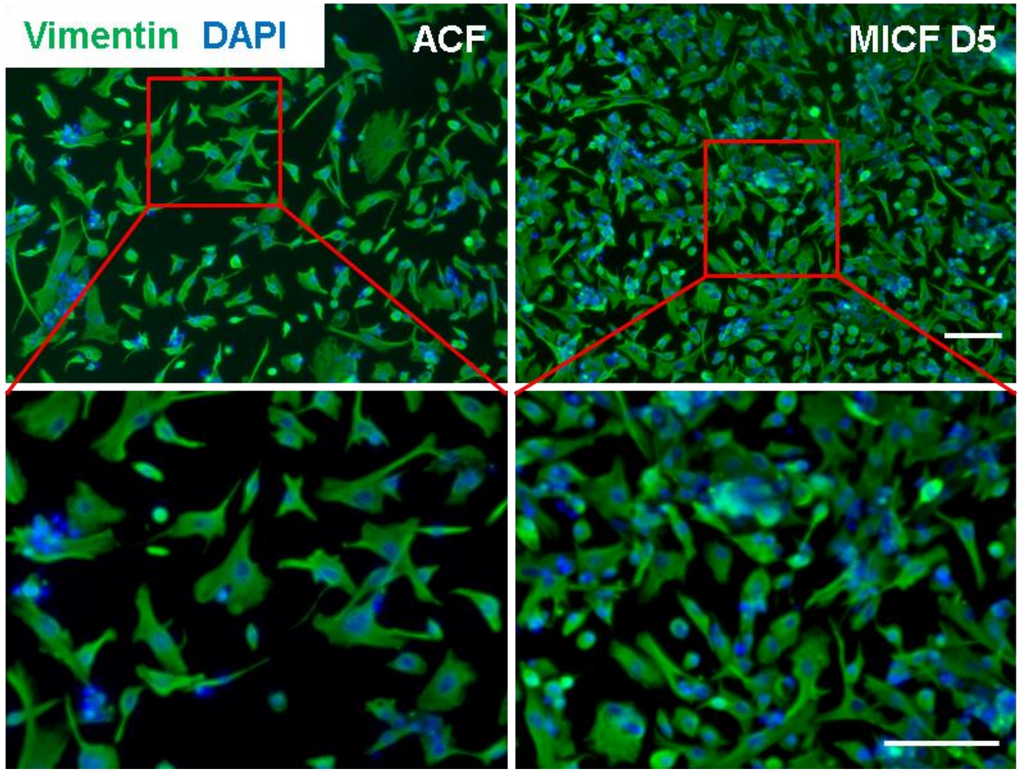

Suppl. Fig. 3B

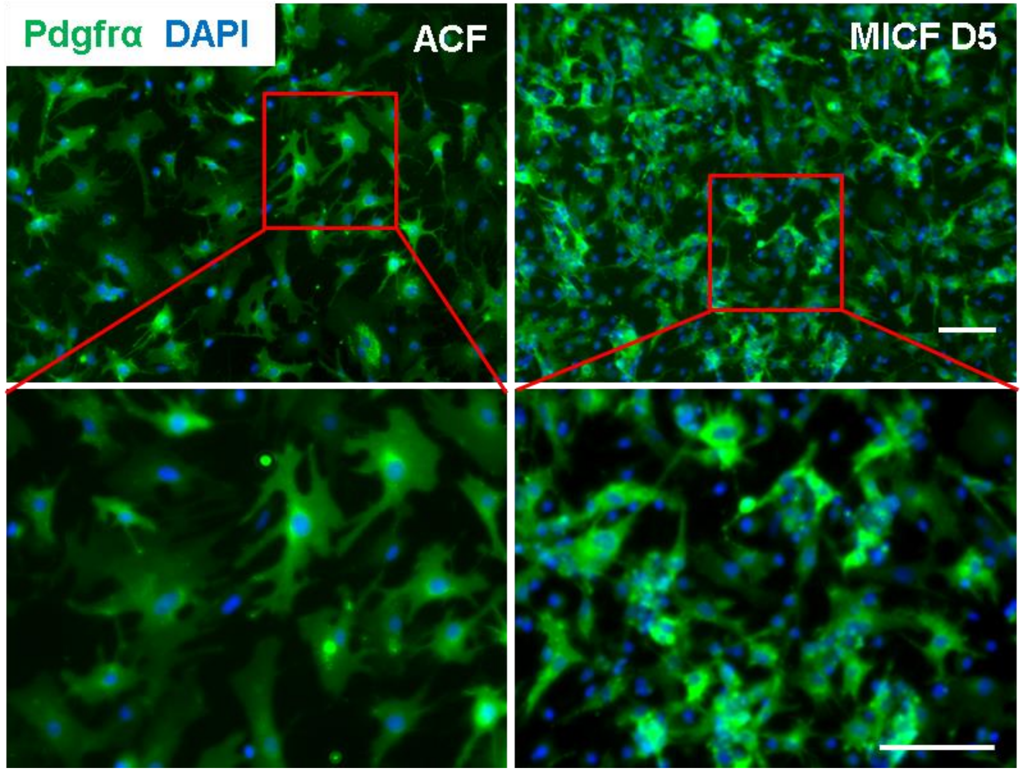

Suppl. Fig. 4A

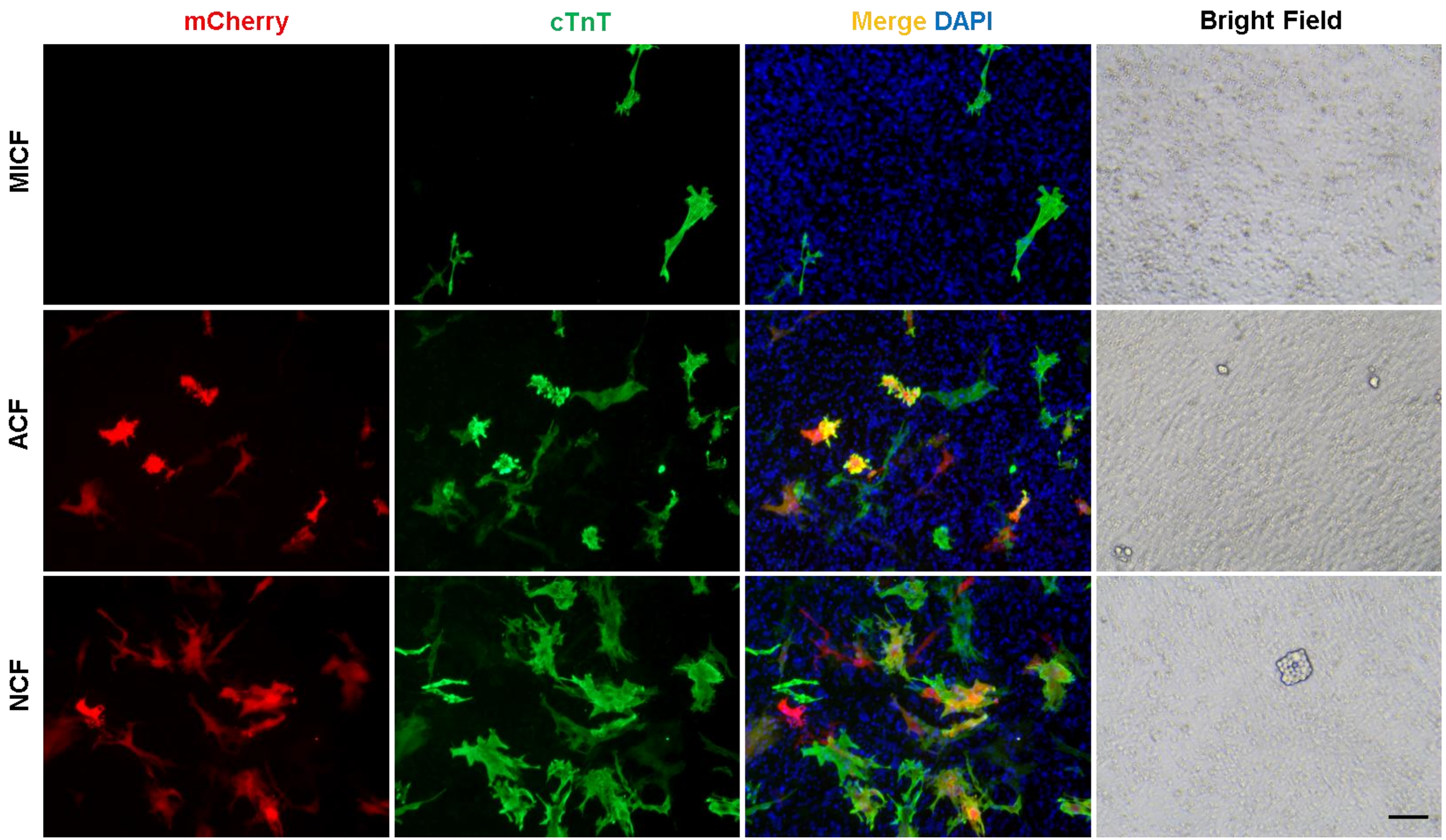

Suppl. Fig. 4B

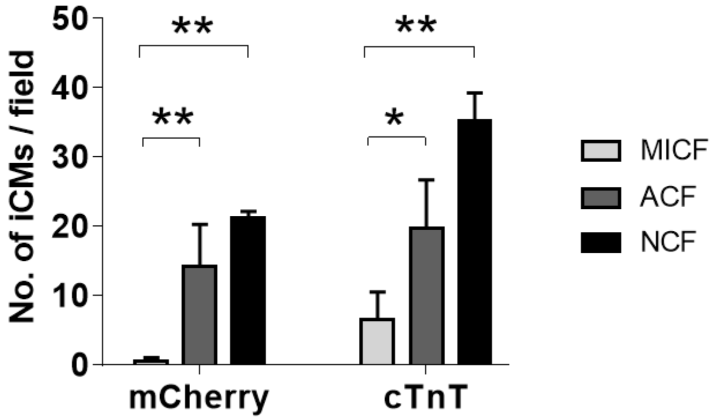

Suppl. Fig. 5

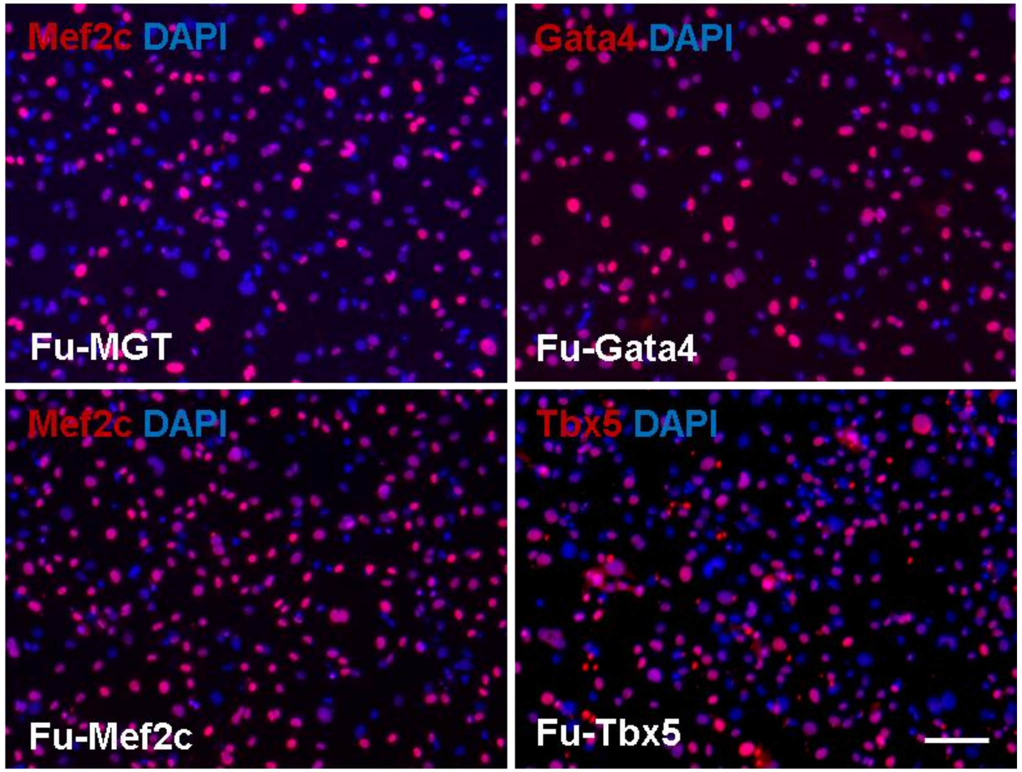

Suppl. Fig. 6A

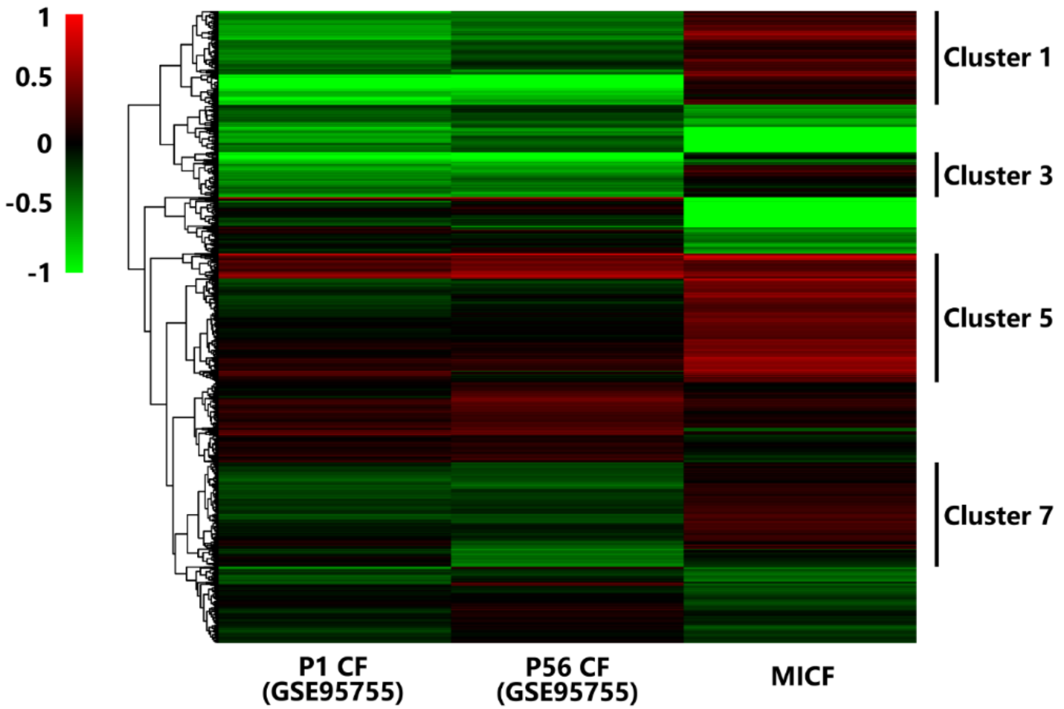

Suppl. Fig. 6B

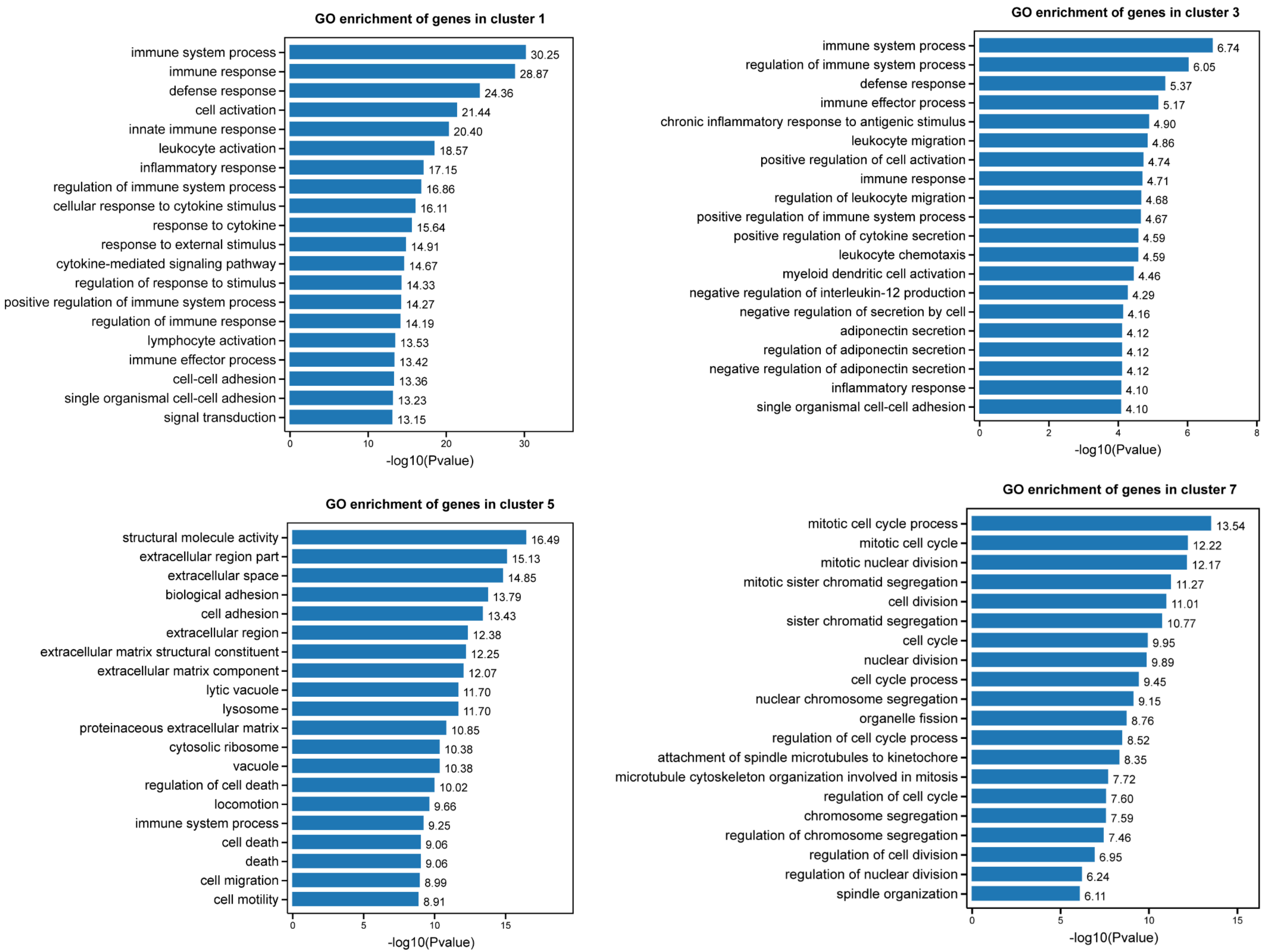

GO enrichment of genes in cluster 1
immune system process 30.25
immune response 28.87
defense response 24.36
cell activation 21.44
innate immune response 20.40
leukocyte activation 18.57
inflammatory response 17.15
regulation of immune system process 16.86
cellular response to cytokine stimulus 16.11
response to cytokine 15.64
response to external stimulus 14.91
cytokine-mediated signaling pathway 14.67
regulation of response to stimulus 14.33
positive regulation of immune system process 14.27
regulation of immune response 14.19
lymphocyte activation 13.53
immune effector process 13.42
cell-cell adhesion 13.36
single organismal cell-cell adhesion 13.23
signal transduction 13.15
0
10
20
30
-log10(Pvalue)
GO enrichment of genes in cluster 3
immune system process 6.74
regulation of immune system process 6.05
defense response 5.37
immune effector process 5.17
chronic inflammatory response to antigenic stimulus 4.90
leukocyte migration 4.86
positive regulation of cell activation 4.74
immune response 4.71
regulation of leukocyte migration 4.68
positive regulation of immune system process 4.67
positive regulation of cytokine secretion 4.59
leukocyte chemotaxis 4.59
myeloid dendritic cell activation 4.46
negative regulation of interleukin-12 production 4.29
negative regulation of secretion by cell 4.16
adiponectin secretion 4.12
regulation of adiponectin secretion 4.12
negative regulation of adiponectin secretion 4.12
inflammatory response 4.10
single organismal cell-cell adhesion 4.10
0
2
4
6
8
-log10(Pvalue)
GO enrichment of genes in cluster 5
structural molecule activity 16.49
extracellular region part 15.13
extracellular space 14.85
biological adhesion 13.79
cell adhesion 13.43
extracellular region 12.38
extracellular matrix structural constituent 12.25
extracellular matrix component 12.07
lytic vacuole 11.70
lysosome 11.70
proteinaceous extracellular matrix 10.85
cytosolic ribosome 10.38
vacuole 10.38
regulation of cell death 10.02
locomotion 9.66
immune system process 9.25
cell death 9.06
death 9.06
cell migration 8.99
cell motility 8.91
0
5
10
15
-log10(Pvalue)
GO enrichment of genes in cluster 7
mitotic cell cycle process 13.54
mitotic cell cycle 12.22
mitotic nuclear division 12.17
mitotic sister chromatid segregation 11.27
cell division 11.01
sister chromatid segregation 10.77
cell cycle 9.95
nuclear division 9.89
cell cycle process 9.45
nuclear chromosome segregation 9.15
organelle fission 8.76
regulation of cell cycle process 8.52
attachment of spindle microtubules to kinetochore 8.35
microtubule cytoskeleton organization involved in mitosis 7.72
regulation of cell cycle 7.60
chromosome segregation 7.59
regulation of chromosome segregation 7.46
regulation of cell division 6.95
regulation of nuclear division 6.24
spindle organization 6.11
0
5
10
15
-log10(Pvalue)

Suppl. Fig. 7

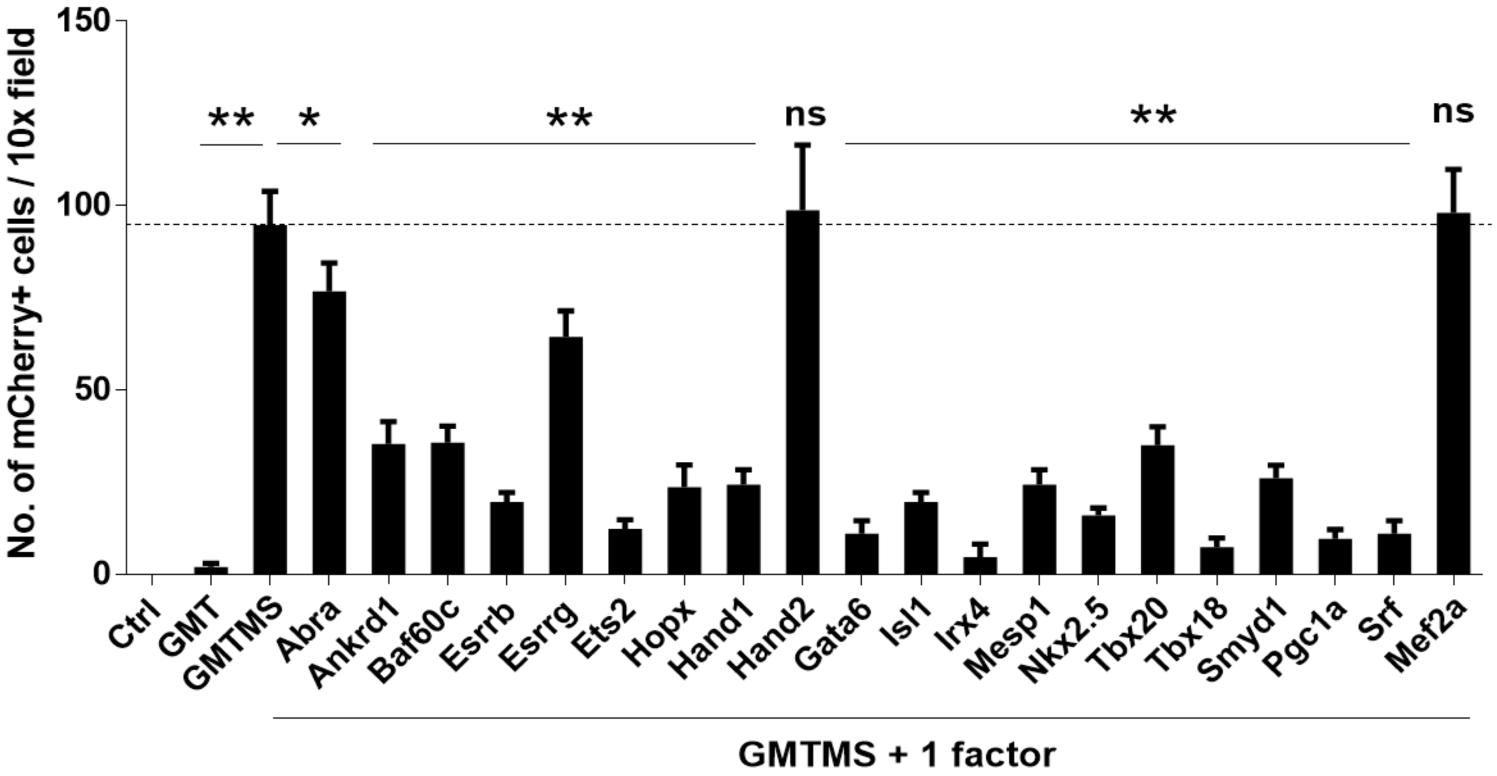
No. of mCherry+ cells / 10x field
150
100
50
0
**
*
**
ns
**
ns
Ctrl
GMT
GMTMS
Abra
Ankrd1
Baf60c
Esrrb
Esrrg
Ets2
Hopx
Hand1
Hand2
Gata6
Isl1
Irx4
Mesp1
Nkx2.5
Tbx20
Tbx18
Smyd1
Pgc1a
Srf
Mef2a
GMTMS + 1 factor

Suppl. Fig. 8A

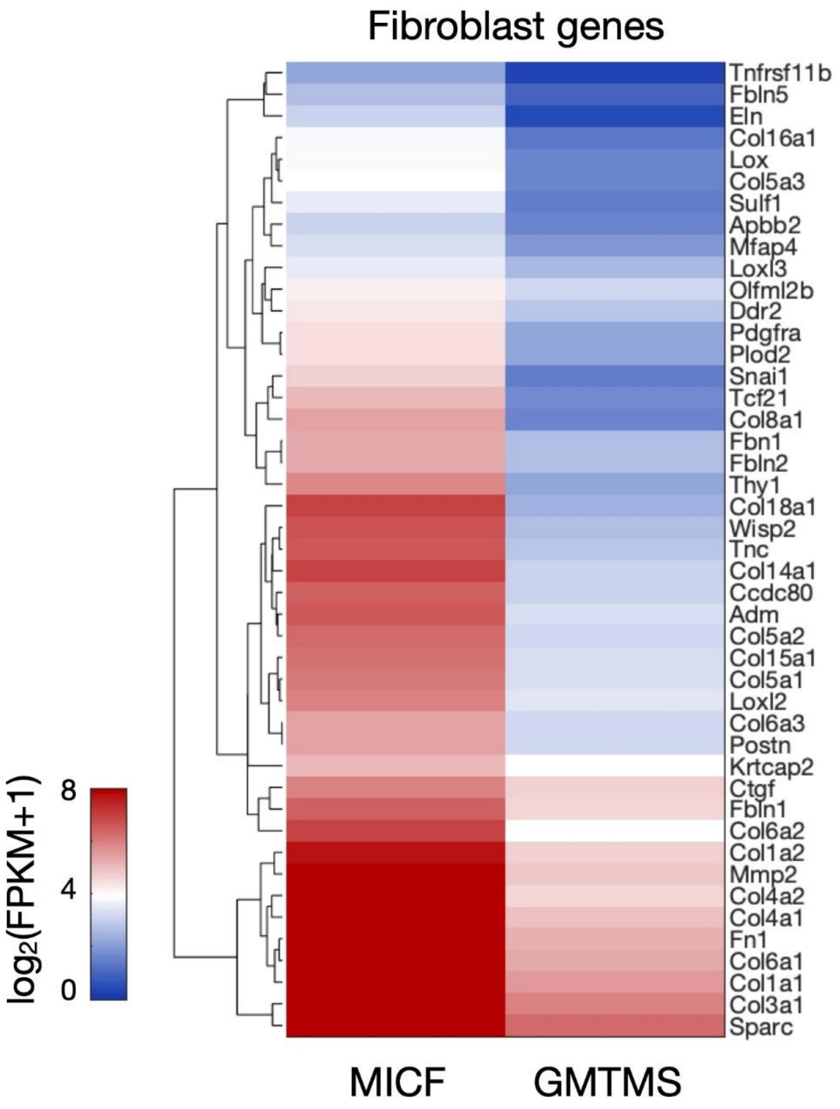

Suppl. Fig. 8B

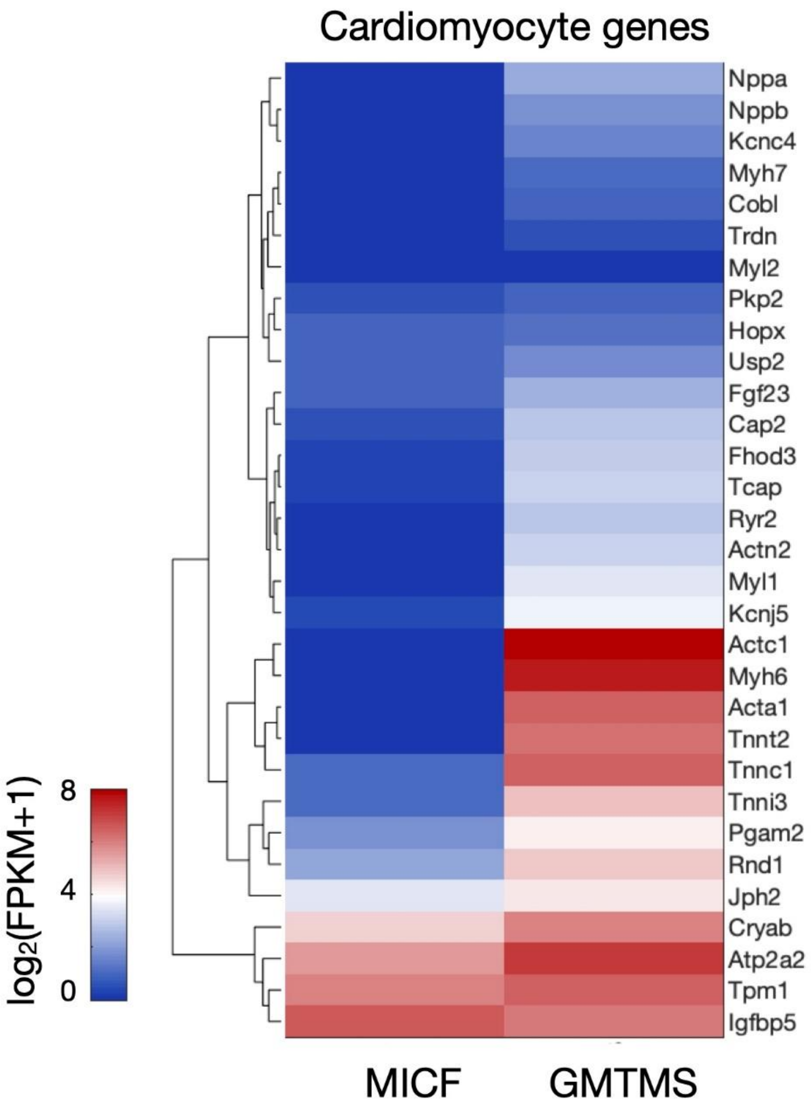

Suppl. Fig. 9A

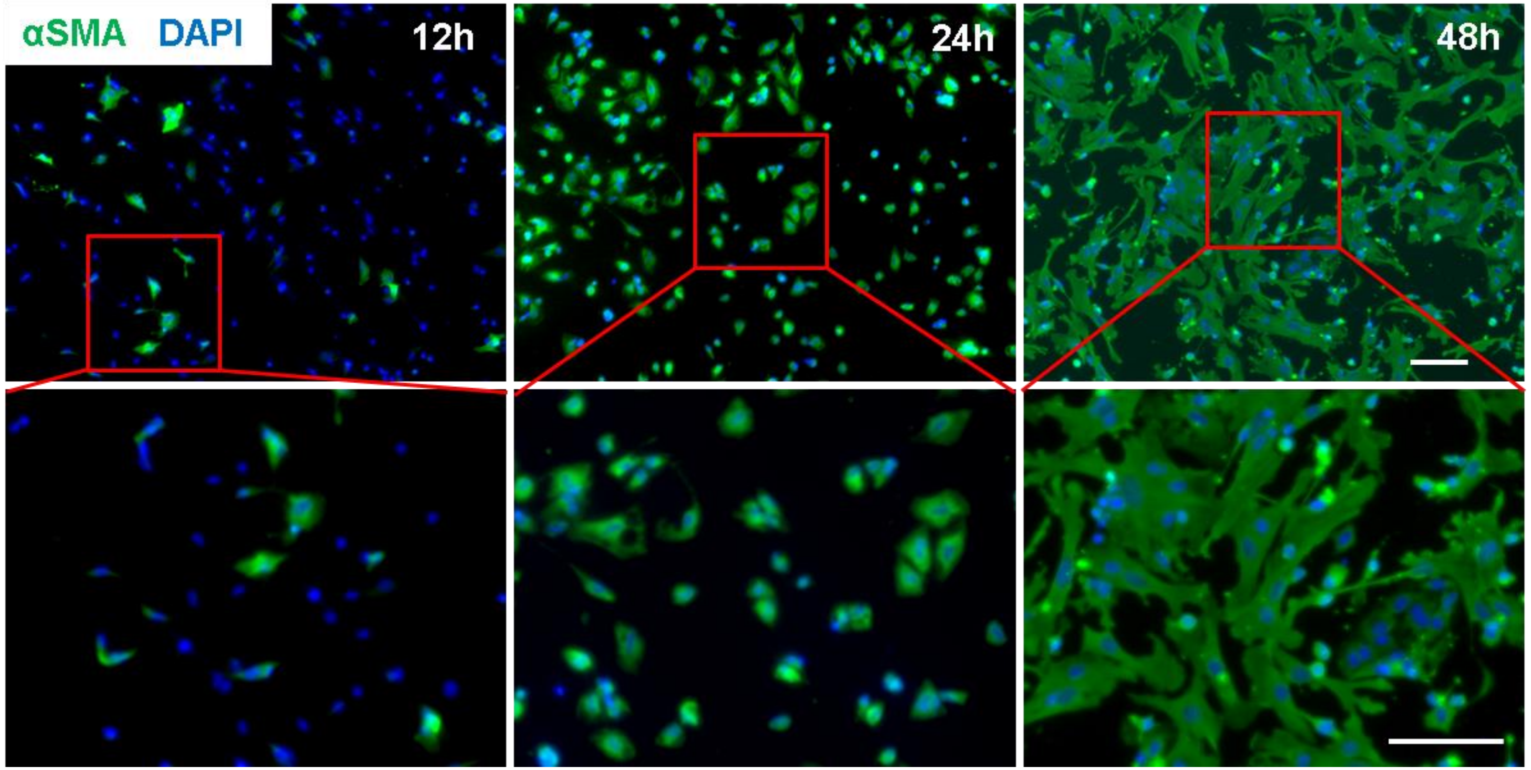

Suppl. Fig. 9B

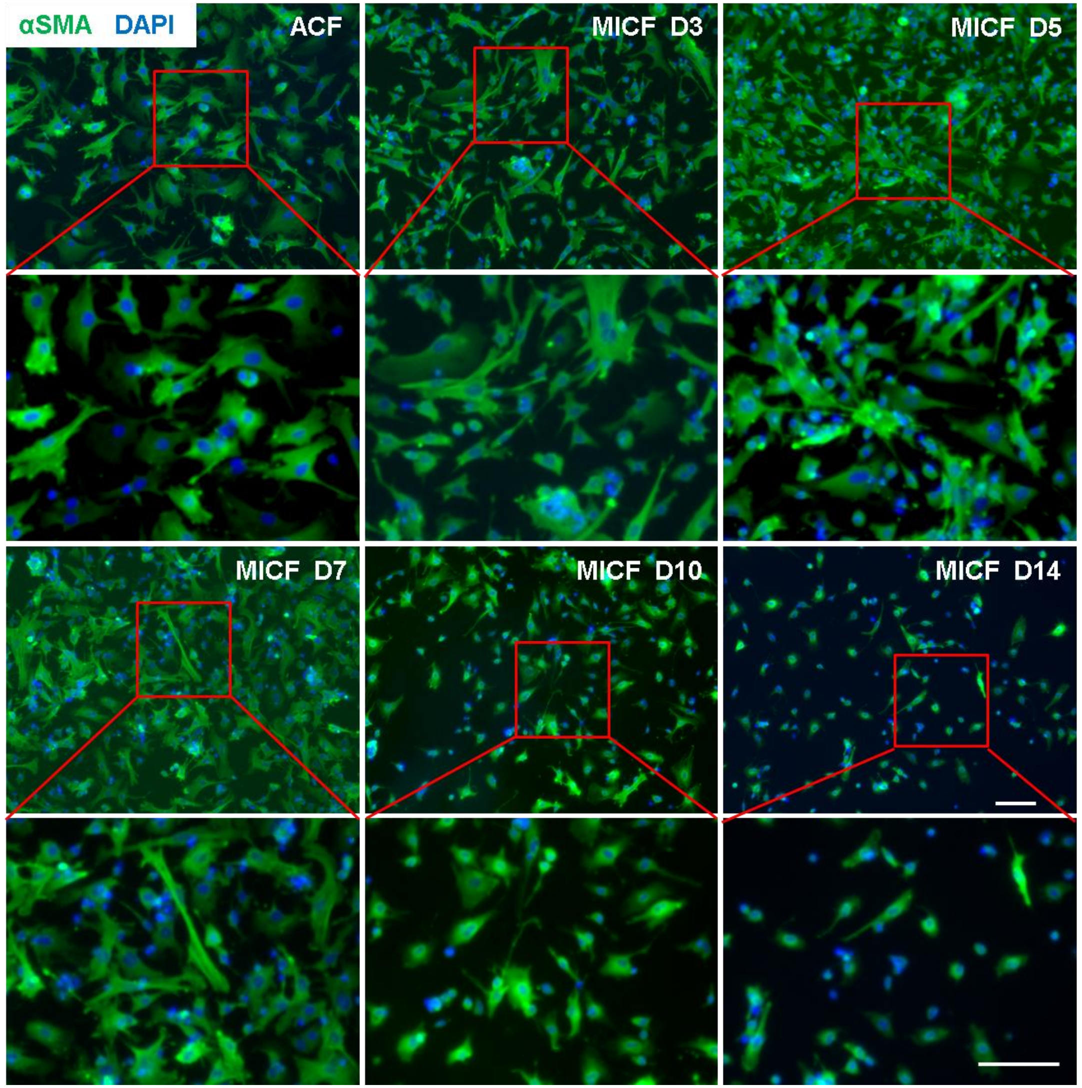

Suppl. Fig. 9C

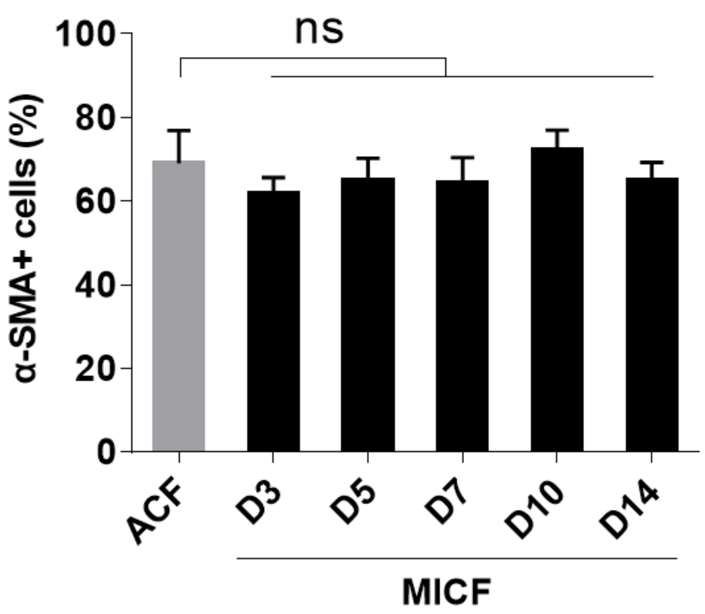

Supplement: Supplementary Figure 1 — Quantity and morphology of MICFs isolated from mice with MI surgery for 3, 5, 7, 10, and 14 days. MICFs without passaging (P0), 48 h post seeding. Scale bars: 100 μm. [file Data_Sheet_1.PDF]
